# Development and feasibility of an intervention featuring individual supported work placements to aid return to work for unemployed people living with chronic pain

**DOI:** 10.1186/s40814-020-00581-6

**Published:** 2020-04-22

**Authors:** Robert Froud, Mary Grant, Kim Burton, Jonathan Foss, David R. Ellard, Kate Seers, Deb Smith, Mariana Barillec, Shilpa Patel, Kirstie Haywood, Martin Underwood

**Affiliations:** 1 Warwick Clinical Trials Unit, Warwick Medical School, University of Warwick, Gibbet Hill Road, Coventry, CV4 7AL UK; 2 Institute of Health Sciences, Kristiania University College, Prinsens Gate 7-9, 0152 Oslo, Norway; 3 Centre for Applied Research in Health, School of Human & Health Sciences, The University of Huddersfield, Queensgate, Huddersfield, HD1 3DH UK; 4 Warwick Research in Nursing, Division of Health Sciences, Warwick Medical School, University of Warwick, Gibbet Hill Road, Coventry, CV4 7AL UK; 5 University/User Teaching and Research Action Partnership, University of Warwick, Coventry, UK; 6 Serco UK & Europe; Employment, Skills and Enterprise, Trigate Business Centre, 210-222 Hagley Road West, Birmingham, B68 0NP UK; 7 University Hospitals of Coventry and Warwickshire, Clifford Bridge Road, Coventry, CV2 2DX UK

**Keywords:** Supported employment, IPS, Chronic pain, Feasibility, Work placements

## Abstract

**Background:**

Working in good jobs is associated with good health. High unemployment rates are reported in those disabled with musculoskeletal pain. Supported employment interventions work well for helping people with mental health difficulties to gain and retain employment. With adaptation, these may be useful for people with chronic pain. We aimed to develop and explore the feasibility of delivering such an adapted intervention.

**Methods:**

We developed an intervention and recruited unemployed people with chronic pain from NHS pain clinics and employment services. We trained case managers to assess participants and match them to six-week work placements in the Midlands and provide ongoing support to them and their managers. Participants attended a two-day work preparation session prior to placement. Outcome measures included quality of life at baseline, six- weeks, 14-weeks, and six-months, and return to work at 14-weeks and six-months. We held focus groups or interviews with stakeholders to examine acceptability and experiences of the intervention.

**Results:**

We developed an intervention consisting of work preparation sessions, work experience placements, and individualised employment support. We enrolled 31 people; 27 attended work preparation sessions, and 15 attended placements. Four of our participants started jobs during the study period. We are aware of two others starting jobs shortly after cessation of follow-up. We experienced challenges to recruitment in one area where we had many and diverse placement opportunities and good recruitment in another area where we had a smaller range of placement opportunities. All stakeholders found the intervention acceptable, and it was valued by those given a placement. While there was some disappointment among those not placed, this group still valued the work preparation sessions.

**Conclusions:**

The developed intervention was acceptable to participants and partners. Trialling the developed intervention could be feasible with attention to three main processes. To ensure advanced availability of a sufficiently wide range of work placements in each area, multiple partners would be needed. Multiple recruitment sites and focus on employment services will yield better recruitment rates than reliance on NHS pain clinics. Maintaining an adequate follow-up response rate will likely require additional approaches with more than the usual effort.

## Background

There are positive associations between good work and good health [1]. Improving work participation for sick and disabled people may improve health outcomes, quality of life, and well-being, and reduce poverty [2]. For many, a job is an essential part of self-identity [2]. Chronic musculoskeletal pain (CMP) is highly prevalent among adults and seldom confined to a single body site [4–7]. This may present substantial challenges for many people [3, 4]. Mental health difficulties are frequently comorbid with CMP, either as a pre-existing condition, or sequelae to the psychosocial and physical challenges associated with living with CMP [5–7].

Individual Placement and Support (IPS) is a highly structured supported employment intervention that is more than twice as likely to lead to employment than traditional vocational rehabilitation for people with severe mental health difficulties [8]. IPS involves close integration of occupational and health services to support return to work (RTW) while people receive any help they need after being placed (i.e. a ‘place then train’ model) [9, 10]. Some trial evidence suggests that integrated clinical and occupational interventions are effective at reducing days lost from work in CMP populations [11]. However, people who are unemployed due to CMP may face different obstacles to gaining or returning to work than those with mental health problems [12]. An intervention to help people with chronic pain to gain or RTW may first need to identify and tackle individual obstacles to working then provide personal and workplace support [13].

Our aim was to explore the feasibility of developing and delivering an intervention featuring individual supported work placements with a case-manager led support package for participants and placement managers. Our objectives were to develop the intervention and explore whether we were able to (1) recruit participants with chronic pain who are unemployed from pain clinics and job clubs and employment services, (2) provide a sufficient number and range of work placements for participants, and (3) deliver the supportive components of the intervention. We also wanted to explore (4) whether the delivered intervention is acceptable to those involved and (5) produce parameter estimates to inform the design of a randomised controlled trial.

## Methods

### Intervention development

Prior underpinning work included a systematic review of supported employment interventions [14], a meta-ethnography on the perceptions of obstacles to RTW of people with chronic pain and employers [15], and in-depth interviews with people off work due to chronic pain, and separately with employers, to capture additional detail about perceptions of obstacles to RTW not covered by our reviews [16]. Using University of Warwick’s University/User Teaching and Research Action Partnership (UNTRAP) group (who have public patient involvement (PPI) training), we also consulted people living with chronic pain, who were unemployed, about their views on both the underpinning work and the feasibility study from point of design to completion.

Our intervention featured specially trained case managers (CMs) to perform work ability assessments with participants, matching them to existing (but modifiable) six-week work placements offered by partners, and providing ongoing support to both participants and workplace managers.

Our placement partners, an NHS Trust, a city council, and a public services provider, each aimed to provide at least 10 work placements of up to 16-hours per week, across a range of roles, settings, and locations.

### Participant recruitment

We recruited unemployed adults with non-cancer chronic pain, who wanted to work but who perceived that pain interfered with ability to work or maintain sustained employment. Participants needed to have been unemployed for at least three-months. However, around one year into the study, we lowered this threshold to one month to facilitate recruitment. We excluded people involved in other work placement schemes, those insufficiently fluent in English to give informed consent and those with ongoing litigation in relation to employment, disability, or accidents involving injury.

We aimed to recruit participants from NHS pain clinics and physiotherapy services, job clubs, organisations providing employment services, a poster campaign, and through local media. Researchers visited each site to explain the nature of the study to staff and provide information materials and response forms, which staff provided to potential participants. Researchers arranged to meet those screened by telephone as eligible, to take written consent, and to collect baseline outcome measures. We aimed to place 30 participants; we have previously found 30 sufficient to gain experience of delivering a complex intervention and adequate to help inform an approximate sample size calculations in future trials [17].

### Training case managers and work preparation

We trained one occupational health nurse and one employment specialist to act as CMs, and we also trained two back-up CMs. Our one-day training package was based on the Psychosocial Flags Framework and a pragmatic understanding of chronic pain and its management, using a biopsychosocial perspective to identify and tackle obstacles to working across the personal, workplace, and social domains [13, 18]. We aimed to provide CMs with the necessary competencies to make an initial assessment, explore work aspirations, identify obstacles to work ability, and collaboratively agree a plan of action.

### The intervention

Recruited participants were allocated to CMs by geographic area. CMs arranged to meet participants to perform the assessment and discuss placements. In the case, no suitable placements could be readily identified, the CM explored whether significant alterations could be made to a placement. If not, the participant was not placed, and the reason for non-placement was recorded.

Prior to placement, participants were required to attend a group work preparation session, which was delivered over two days and comprised practical advice about working with chronic pain including self-reflection, myth busting, and work adaptations. The material used in the work preparation session was largely based on work and health evidence from the literature [13, 18]. Any placement-specific training required by the placement provider was given by the placement provider. For example, participants with an NHS Trust placement were required by the Trust to do a further two-day training session.

The CM collaboratively facilitated the agreement of a work plan with the placement manager and participant and provided regular and as required support to the participant and placement manager (either by telephone or face-to-face).

### Outcome measures

We assessed the feasibility of administering the outcome measures and collected the data shown in Table 1. A study administrator sent out follow-up questionnaires and made telephone calls to non-responders.

**Table 1:** Outcome measures, scale details and data collected by time-point

Outcome measure	Scale details	Measurement time-points
Demographic data on gender, marital status, ethnicity, locations of chronic pain using pain drawing, duration of chronic pain symptoms, concomitant health symptoms, highest level of education, length of time out of work, and length of time looking for work	Categorical/quasi-continuous	Baseline
EQ-5D-5L [43]	0 to 1, where higher scores indicate greater health	Baseline, six-weeks, 14-weeks, six-months
Hospital and Anxiety Depression Scale (HADS )[44]	0 to 21 on each scale, where higher scores indicate greater anxiety or depression	Baseline, six-weeks, 14-weeks, six-months
Confidence in working	11-point numerical rating scale (NRS), where higher scores indicate greater confidence	Baseline, six- weeks, 14-weeks, six-months
Work ability [45]	11-point NRS, where higher scores indicate greater perceived work ability	Baseline, six-weeks, 14-weeks, six-months
Current pain intensity [46]	11-point NRS, where higher scores indicate greater pain intensity	Baseline, six-weeks, 14-weeks, six-months
Pain intensity over past week [46]	11-point NRS, where higher scores indicate greater pain intensity	Baseline, six-weeks, 14-weeks, six-months
The Warwick-Edinburgh Mental Wellbeing Scale (WEMWBS) [47, 48]	14 to 70, where higher scores indicate greater well-being	Baseline, six-weeks, 14-weeks, six-months
PROMIS short-form questionnaire for fatigue (8a) [49]	8 to 40, where higher scores indicate greater fatigue	Baseline, six-weeks, 14-weeks, six-months
PROMIS short-form questionnaires for pain interference (8a) [50]	6 to 30, where higher scores indicate greater pain interference	Baseline, six-weeks, 14-weeks, six-months
Satisfaction with placement	A 5-item scale from ‘very dissatisfied’ to ‘very satisfied’	Six-weeks (and 14-weeks/six-months if job offer accepted)
Work Role Functioning Questionnaire (WRFQ) [27, 51]	0 to 100, where higher scores indicate better work functioning	Six-weeks (and 14-weeks/six-months if job offer accepted)
Psychosocial Aspects of Work (PAW) [52]	Job satisfaction, 7 to 35; social support, 4 to 20; mental stress, 4 to 20; where higher scores indicate greater domain perceptions	Six-weeks (and 14-weeks/six-months if job offer accepted)
RTW Self-Efficacy Scale [53, 54]	2 to 10, where higher scores indicate greater self-efficacy	Six-weeks (and 14-weeks/six-months if job offer accepted)
Pain interference	A 5-item scale from ‘not at all’ to ‘extremely’	Six-weeks (and 14-weeks/six-months if job offer accepted)
Whether work makes pain worse	A 5-item scale from ‘not at all’ to ‘extremely’	Six-weeks (and 14-weeks/six-months if job offer accepted)
Job offers	Number of job offers (from any source)	14-weeks and six-months
Details of accepted job offers	Number of job offers accepted, job title of accepted offer, start date, and work pattern	14-weeks and six-months
Adverse events, serious adverse events	Case report details	Point of occurrence
Healthcare referrals	Case report details	Point of occurrence

### Process evaluation and analysis

We did a mixed-methods process evaluation within a quasi-experimental design to assess feasibility of delivering the intervention and describe transferable principles about the intervention to inform its implementation on a wider scale [19–22]. We considered context, reach, dose delivered, dose received, and recruitment [23].

CMs recorded details of interactions, activities, and their durations. Participants self-reported attendance so that we could describe dose received. We did in-depth qualitative interviews with consenting placement managers, case managers, and recruiters. We analysed field notes from observations. We held focus groups with participants who were placed and also with participants who we could not place. We did individual interviews if people could not attend the group session. We reimbursed participants’ travel expenses and provided a £20 high street voucher in gratitude for their time. Data from focus groups and interviews were audio recorded, transcribed verbatim, and analysed thematically [24]. We identified themes and similarities and differences within and between participants (without using any formal analytical framework). Themes were coded using the QSR NVivo 12 software (QSR, Victoria). Verbatim comments are presented that support and add context to the written summary.

We summarised quantitative data from the study using descriptive statistics. We estimated change scores to within a 95% confidence interval. We mapped our EQ-5D-5L (Table 1) data onto the 3L valuation set [25] and reported these predicted values, conditional on age and gender, using the Stata package *eq5dmap* [26]. For HADS, PROMIS, and PAW outcome measures (Table 1), we imputed subscale averages in the cases of single items missing, and dropped whole cases in the case of multiple missing items, or in the case of the WRFQ (Table 1), if participants checked the ‘does not apply to my job’ response for over 20% of items [27].

### Ethics

The study received favourable opinion from the London–Stanmore National Research Ethics Committee (17/LO/0919) on July 19, 2017.

## Results

We recruited from three NHS pain clinics from September 2017; one was a hospital clinic run by medical doctors and focused on medical intervention, and two were hospital clinics with multidisciplinary teams that were led by medics, with pain management services coordinated by physiotherapists, and had the input of a clinical psychologist. From December 2017, we recruited from a centre for council-run services for people looking for work. From January 2018, we displayed posters across 49 libraries, 47 GP practices, 11 supermarkets, 13 careers services, three job clubs, and on four pain-related charity websites, where we invited people to contact us directly. From May 2018, we added a privately held organisation, which provides local employment services for people looking for work.

**Fig. 1:**
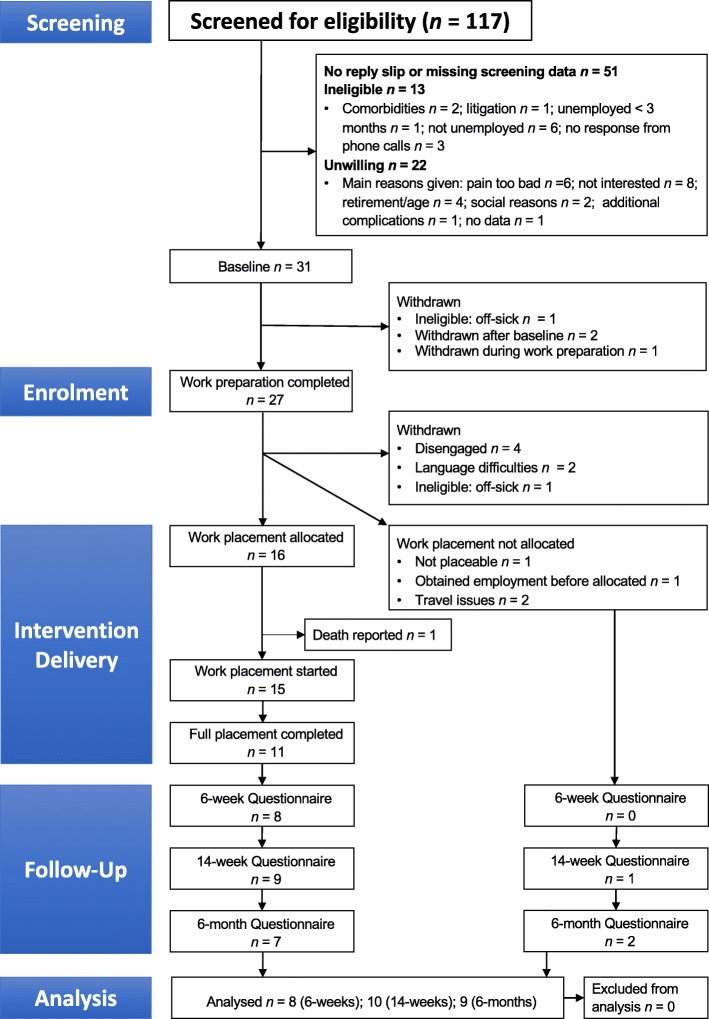
Consort diagram. The diagram shows the flow of participants through the study

**Fig. 2:**
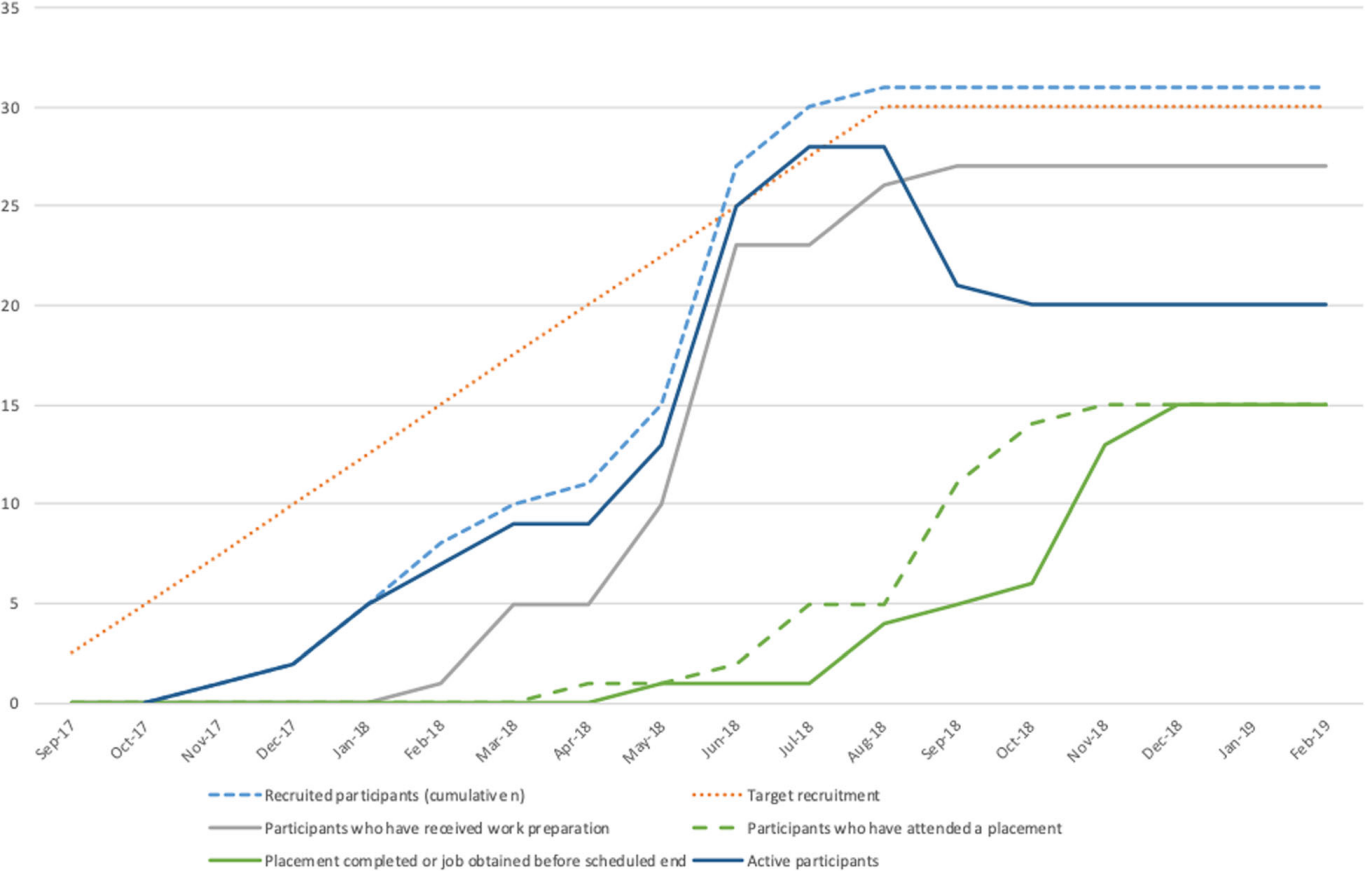
Recruitment, work preparation, and placement completion. The figure shows recruitment, rates of work preparation, and work placement completion by time

We screened 117 people for eligibility (Fig. 1). We recruited participants between September 2017 and August 2018 to permit a 6-month follow-up prior to the planned end date in February 2019. Figure 2 shows timescales of recruitment, delivery of work preparation, and placement completion. We received expressions of interest from 33 eligible people. Three people screened in NHS settings returned forms, the remainder coming from non-NHS sites and direct contact (following responses to posters, etc.). The recruitment distribution was approximately 10% from NHS sites, 58% from non-NHS sites, and 32% from direct contact.

We recruited 31 participants. Twenty-two participants (71%) had pain in two or more body areas (Table 2). All participants had previously worked. All but six participants reported additional health concerns including mental, cardiovascular, rheumatological, and musculoskeletal health, and also alcoholism. One group work preparation session ran in Birmingham and three in Coventry. Two people were found to be ineligible post-recruitment due to being off-sick rather than unemployed. Two were unable to be placed due to language/learning difficulties. Three withdrew after baseline, one citing multiple dental operations, one who did not want to participate, and one on the first day of work preparation (no reason given; however, the participant had been unsure about starting the placement and by lunchtime of the first day had decided not to continue).

**Table 2:** Baseline sample characteristics (*n*=31)

Characteristic	
Gender, *n* (%) female	18 (58)
Age, mean (SD)	50.9 (10.5)
Marital status, *n* (%)	
Single (never married)	16 (52)
Married	9 (29)
Separated or divorced	6 (19)
Areas of pain, *n* (%)	
Lower back	20 (65)
Back or neck	23 (74)
Head	6 (19)
Chest	1 (3)
Abdominal	1 (3)
Peripheral	7 (23)
Ethnicity, *n* (%)	
White	20 (65)
Black	2 (6)
Asian	4 (13)
Mixed	5 (16)
Highest education, *n* (%)	
Degree/higher degree	5 (16)
Higher education, A-level/equivalent	8 (26)
GCSE/O-level/equivalent	13 (42)
Vocational/work-related	4 (13)
No qualification	1 (3)
Years in pain, median (IQR)	11.8 (2.5 to 18.0)
Years out of work, median (IQR)	6.3 (0.5 to 8.0)
Years looking for work, median (IQR)	4.4 (0.3 to 3.0)
Perceived work confidence, mean (SD)	6.2 (2.4)
Perceived work ability, mean (SD)	5.5 (2.4)
EQ-5D health today, mean (SD)	53.9 (19.6)
EQ-5D 5L mapped to 3L value set, mean (SD)	0.53 (0.22)
HADS anxiety, mean (SD)	8.4 (4.4)
HADS depression, mean (SD)	6.8 (3.2)
NRS current pain, mean (SD)	5.9 (2.3)
NRS pain over past week, mean (SD)	6.4 (2.4)
WEMWBS, mean (SD)	23.7 (5.7)
PROMIS SF, fatigue; mean (SD)	27.9 (8.2)
PROMIS SF, pain interference; mean (SD)	28.5 (8.3)

We experienced delays in contracting, achieving a final signed contract with all research partners after approximately 20-months. Our partners offered 29 job placements. Additional file 1 shows the allocated placements and the available placements provided by our public services provider partner, our council partner (10 across five sites with one becoming withdrawn), and our NHS Trust partner (10 to be selected from 22 possible roles). As we had a less diverse range of placements in Coventry, the University of Warwick also provided a placement to help improve the range in that area. In total, there were 19 placements offered in Birmingham and surrounding areas and 10 placements in Coventry.

We provided the work preparation session to 27 people. We ran five two-day group work preparation sessions where group sizes ranged from two to 12 people (with a median of five people). An additional single one-to-one session was provided at a participant’s home. While we had intended for all preparatory sessions to be delivered in group settings, we made a reactive alteration for the last participant who had not attended the final timetabled group session.

The mean time between recruitment (and baseline measurement) and the start of job placement or the point at which a participant accepted a placement was 94.8 days (SD = 35.5).

### Placement completion and attendance

Fifteen of the 31 people recruited (48%) were matched and attended a supported work placement (Fig. 1). All but four participants completed the full six weeks. Reasons provided for non-completion included moving into paid employment (PP02), personal difficulties (PP19), not being happy in the placement (PP22), and health reasons (PP30). All placement work was completed between Monday and Friday, with an average total placement hours of 52.5 (SD = 32.9) over 5.5 weeks (1.0). The average number of hours worked per week was 9.4 (5.2) with a range of 1.3 to 16.7 hours.

Four participants who were offered placements disengaged. Two wanted placements in Coventry when only Birmingham placements were available. One obtained employment before case management started.

Table 3 shows within-group change scores since the start date of placement or the date at which it was decided a participant could not be placed. Table 4 shows work-related outcomes relating to placement. Table 5 shows reported employment offers, and one formally reported acceptance at follow-up (PP11).

**Table 3:** Follow-up time-points. Crude score changes from baseline (follow-up, start date)

	six-weeks (95% CI)	*n*	14-weeks (95% CI)	*n*	six-months* (95% CI)	*n*
Perceived work confidence	− 0.4 (− 2.9 to 2.0)	7	− 1.1 (− 4.0 to 1.8)	10	− 3.0 (− 6.2 to 0.2)	9
Perceived work ability	0.4 (− 2.3 to 3.1)	7	− 0.1 (− 2.3 to 2.5)	10	− 0.8 (− 3.7 to 2.1)	9
EQ-5D health today	4.8 (− 10.6 to 20.1)	8	− 0.2 (− 13.5 to 13.1)	10	− 6.9 (− 24.8 to 11.8)	9
EQ-5D 5L ➔ 3L^†^	− 0.06 (− 0.19 to 0.07)	8	− 0.08 (− 0.24 to 0.08)	10	− 0.06 (− 0.26 to 0.15)	9
HADS anxiety	− 0.9 (− 2.7 to 0.9)	8	0.2 (− 0.8 to 1.2)	9	0.1 (− 4.3 to 4.5)	9
HADS depression	1.0 (− 1.5 to 3.5)	8	2.6 (− 0.3 to 5.4)	9	2.2 (− 1.0 to 5.4)	9
NRS current pain	− 0.6 (− 3.2 to 1.9)	8	− 0.6 (− 2.2 to 1.0)	10	− 0.8 (− 2.7 to 1.1)	9
NRS pain over past week	− 0.6 (− 2.4 to 1.2)	8	0.2 (− 2.1 to 2.5)	10	− 0.7 (− 3.6 to 2.3)	9
WEMWBS	− 0.8 (− 5.0 to 3.5)	8	− 1.6 (− 5.2 to 2.1)	9	− 1.7 (− 6.4 to 3.1)	9
PROMIS SF fatigue	− 0.6 (− 9.8 to 8.5)	8	− 2.3 (− 7.1 to 2.4)	9	− 1.6 (− 9.5 to 6.4)	9
PROMIS SF pain interference	3.1 (− 4.1 to 10.4)	8	0.0 (− 6.1 to 6.1)	9	1.9 (− 6.3 to 10.1)	9

Negative magnitudes represent decreases in score and positive magnitudes represent increases in score. Start date is defined as the start of placement, for those placed, or the time at which it was decided that a person could not be placed, for those not placed

*n.b final time-point was shorter than six-months for some participants to maximise data collection and longer than 6 months for others due to failure to return on time. The median time from start date to final follow-up was 211 days (IQR 175 to 231)

^*†*^We mapped EQ-5D 5L scores onto the 3L value set as per the NICE position paper recommendation (see the “Methods” section) [25]

**Table 4:** Work-related outcomes relating to the placement

Outcome	6-week scores	*n*
Satisfaction with placement, *n* (%)		8
Very satisfied	4 (50)	
Satisfied	2 (25)	
Neither satisfied nor dissatisfied	0 (0)	
Dissatisfied	1 (12.5)	
Very dissatisfied	1 (12.5)	
Psychosocial aspects of work, mean (SD)		
Job satisfaction	26.0 (9.4)	8
Social support	15.4 (5.9)	8
Mental stress	14.4 (5.4)	8
RTW self-efficacy scale, mean (SD)	7.7 (2.3)	8
Work functioning role questionnaire, mean (SD)	55.0 (30.7)	6
How much did pain interfere with your work? *n* (%)		8
Not at all	1 (12.5)	
A little bit	0 (0)	
Moderately	4 (50)	
Quite a lot	3 (37.5)	
Extremely	0 (0)	
How much did work make your pain worse? *n* (%)		8
Not at all	0 (0)	
A little bit	1 (12.5)	
Moderately	3 (37.5)	
Quite a lot	4 (50)	
Extremely	0 (0)

**Table 5:** Job offers, jobs accepted, and jobs (i.e. paid employment) started

	14-week scores	*n*	6-month scores	*n*
Number of job offers reported	2	8	3	9
Number of job offers accepted	0	1^¶^	2	3*
Number of new jobs started	0	0	1	2*

^¶^*i.e.* one person received two offers at 14 weeks

*i.e. those who had offers to accept and of those who had accepted offers, respectively

In a process evaluation interview, one participant described attending a placement but leaving after two and a half weeks for paid employment (PP02). One participant obtained full-time employment after attending the work preparation but prior to being placed (PP12). However, neither returned the final follow-up outcome measures. At six-months, one participant reported receiving an offer but had not then started work (PP21, Table 5). We learned that this person started work after their follow-up was returned. We similarly learned from a CM that another person who had not returned a follow-up had obtained employment (PP09). The participant who left having done half a day of the work preparation also later gained employment (PP03). In total, we are aware of six participants (19%) obtaining employment.

### Adverse events and serious adverse events

Two adverse events were reported. One participant developed an unrelated health problem but following its investigation completed the work preparation and two- weeks of a placement. Another experienced increased pain following an outdoor activity that was performed as part of their placement. There was one death reported by email from the email account used to send a reminder of placement attendance (unrelated to the intervention).

### Recruitment context and perspectives

At each site, researchers met with recruiters to explain the study and inclusion criteria. At non-NHS sites, information was provided about the definition and nature of chronic pain. In the NHS, potential participants were identified in pain clinic consultations or by physiotherapists working in pain management or rheumatology services. Outside the NHS, employees from employment services sites sent information out to people they identified as potentially suitable from databases.

We interviewed four people from three recruitment sites. Recruiters reported finding the paperwork and process straightforward. At one NHS pain clinic, it was suggested that many people were either retired or employed and therefore ineligible. More generally, it was noted that the reason for attending hospital rather than being managed in primary care was because people had complex medical problems (Table 6, quote 1). At one NHS pain clinic, it was suggested that a heavy clinical workload and involvement in a high number of research projects might contribute to a reduced capacity for assisting with recruitment.

**Table 6:** Exemplary quotes from themes emergent from interviews and focus groups

Quote number	Participant	Quote
1	PESI02 (pain consultant)	“At least 60 to 70% [sic] of the cohort of people we come across have severe mental health issues”
2	PESI05 (rheumatology physiotherapist	“… all of them will say that on the good days they feel perhaps that they could do something from a work point of view. But then it’s also, you know, they don't know when these bad days are going to be, and it’s that fear of not being able to do it on the, the bad days and the impact that, that has on their condition, and how that would be viewed by the, the potential employer”
3	PESI11 (recruiter from a non-NHS site)	“Then, there was a few people concerned about how it would affect their benefits. … That was, that was a common theme for people that said, ‘No’ or, you know, weren’t interested, because they, I think, for some of them, they felt that if they were on a placement for 16-hours a week, would then, DWP try and make them work 16-hours a week when they, they didn’t feel they were able to? So they couldn’t sort of differentiate when I was trying to say, ‘But this will, the study will help us identify if you can work those hours. Or it might identify that you can’t, but you can work eight.’ But you might not be able to work 16 but some of them just weren’t willing to, sort of give it a go. Yeah. … That was a big thing for people, the benefits.”
4	PESI07 (placement manager)	“But personally, I felt that I was helping someone ... it's not relevant to this survey [sic] but by my wife has been medically retired for very similar reasons, so I understand my wife’s frustrations. But then also, also being able to bring that into, into work and if, you know, helping somebody that's got something fairly similar get them back into a work ethic and maybe that will, er, leapfrog them into full time work … “
5	PESI10 (placement manager)	“… obviously we would like to help out if we can, but we thought, ‘Actually, we need some resource as well,’ and, and so I think, you know, there was a benefit in it for us as well.”
6	PESI10 (placement manager)	“And, and, if it helped her ultimately, yeah ...glad to be involved. But, um, you know, from our perspectives the process was just too short to warrant the amount of training”
7	PESI09 (placement manager)	“She didn't talk about anything other than her illness quite voiceably most of the time that she was here. And so that was a very negative impression that she was giving which meant she got quite a lot of negative responses.”
8	PESI09 (placement manager)	“I think we were surprised funnily enough how much they needed to move around. I think we were expecting people that needed to sit and do sedentary tasks, and actually a lot of them, “No, no, actually my back gets really stiff if I sit, so I need something that's going to involve me moving around”
9	PESI08 (placement supervisor)	“He started, so, so. He was getting more and more in, as if he was, sort of, really getting into what he was doing. 'Cause he was really doing a good job. And I mean, we were getting the public saying, ‘Who's he?’”
10	PESI08 (placement supervisor)	“He was very much in a shell to start off with but of course, by coming in with us at break times, which I think was probably quite a good thing to do for him ... he started to talk, people were talking to him.”
11	PP13 (participant)	“And knowing that we are here and we are trying to fight against all that things. It’s very good, it makes me feel like I’m not alone, there’s a lot of people in the same boat.”
12	PP17 (participant)	“I think we’re all in agreement that the two-day course was, I found, I thought it was brilliant. And it was, like, tailored to us. But one of the biggest things was when we were all explaining our own individual selves, everybody in the room’s going ...”Yeah, it’s true.” Because they understand.
13	PP02 (participant)	“she went above and beyond her job by finding out what my specific condition was. And because it was something very different to what a lot of people have then it made it all the more different to me ...”
14	PP20 (participant)	“And the, fortunately I’m very lucky, *** [placement provider] spoke to someone in the IT audits department and they absorbed me there, and still ongoing in the process, I’ve not completed my six weeks. I think I’ll complete it almost three weeks now. And I really enjoyed the place and the work over there, and actually I thought that I would be underutilised since it’s work experience and with no pay, they might be giving me simple tasks like that. But seeing my potential over there they took me on board and I’m going to do an audit for them.”
15	PP01 (participant)	“I enjoyed all the groups ([work preparation)] and doing it, but quite disappointing that, that I didn’t come away with anything, I didn’t even get a, a placement.”
16	PP13 (participant)	“So for me it was a, a great experience even if they didn’t put me, um, into training [placement], because the training [placement] they wanted me to put me was in Birmingham but because in Birmingham I will take one hour and 30 minutes travelling and come back, and I couldn’t because I’m a single mum with my son.”
17	PP02 (participant)	“And I think with the study as well, because obviously it put me back in touch with, like, people at work, not just social, it gave me back my management confidence, to just go into work ...”

Site recruiters reported potential factors that may have influenced potential participants’ decisions for not joining: perceived uncertainty about work requirements and self-doubt about being able to fulfil the commitment (quote 2), a lack of confidence following being out of work, unpredictability of pain, concerns about ability to travel to and participate in work preparation and placements, family commitments, concerns about finances or becoming worse off, and losing benefits or fear that through completing a placement for 16-hours a week; then, they would be perceived as able to work and be moved off disability benefits but still unable to find paid employment (quote 3).

One recruiter suggested a distinction between those who were desperate to RTW and therefore willing to try the placement and those who may not really want to return to work and were therefore more reluctant to be involved. At non-NHS sites, a potential for misunderstanding the term 'chronic' as meaning 'severe' (in relation to pain) was noted. It was suggested that definitions of terms should be provided with the inclusion criteria.

### Placement provider perspectives

We interviewed three people representing all three placement providers. One initially reported difficulty in providing 10 placements. It was suggested that related challenges could be improved through the initial agreements being not only with a senior level manager but also those who would be involved in providing placements.

Providers reported that for roles in larger organisations, the processes for enabling someone to start work (e.g. induction into IT systems, data protection training) are time-consuming, and it was suggested that this might have been better addressed in advance of a placement starting.

One provider offered 10 placements in a variety of settings around Birmingham. However, only one of the placements was taken due to limited recruitment in the area. Another partner offered a wide range of placements, but due to limited recruitment in the area, only four were used. One provider suggested that expectations, communication, and timescales would have to be managed more effectively if a larger scale study takes place.

### Placement manager perspectives

We interviewed six placement managers. One manager raised concern that involvement had been agreed with higher level management, but that it would have been helpful if placement managers had also been involved earlier to permit orientation of other staff who had reported concern about the placement activity. In this case, the participant had left the placement early.

There was some suggestion that managers’ personal experiences may have influenced their approaches or perceptions of the intervention (quote 4). Some thought involvement provided a valuable resource (quote 5). However, the time it takes to train and support someone was reported as a concern by others (quote 6).

Generally, the level of contact from CMs was thought appropriate. Managers found it helpful when CMs provided background information about participants’ abilities and limitations so that they knew what to expect and could think about how best to work with them. In general, managers found the initial face-to-face meeting with the participant and case manager useful.

Most managers reported participants disclosed information about pain to them, but not to peers in the workplace, although there was an exception (quote 7). Some managers described creating a bespoke role to suit the participant’s ability and adapting roles or tasks. One reported being surprised how much participants needed to move (quote 8).

Generally, managers were satisfied with quality of work (quote 9). There was an example of a case in which the placement provider felt that a participant had the capacity to fulfil all aspects of a role but another who needed more direction than anticipated. One manager noted thinking that participants had benefited from social interaction with colleagues and getting back into the routine of work (quote 10).

One manager thought the placement was a good opportunity to see what participants were capable of and noted then being keen to offer employment. This manager suggested that there were potential benefits to both parties of a 'zero-hours' contract, in that these could be flexible and the participant could choose to work when they felt well. Another noted not being likely to employ someone with chronic pain due to physical demands of the work in question. Perceptions of reasons for non-completion included leaving for a paid job, personal problems, and not being a suitable match for the placement.

### Case manager perspectives

We interviewed all three CMs. They found the process and paperwork acceptable and the training useful. Most assessments were performed face-to-face. One CM who had made contact over the phone reported that face-to-face assessments were preferable. Perceptions of participants’ obstacles included cost, effort, and time of travelling to the placements; concerns about manual handling or not being able to fulfil placement role; childcare issues; and concern about how others in the workplace might perceive them because of pain and fatigue not being visible. Additional issues such as learning difficulties, chronic fatigue syndrome, or other comorbidities were thought to complicate some cases.

Matching participants to placements was challenging because there was good recruitment in Coventry but few suitable placements and poor recruitment in Birmingham but many placements. CMs thought there was insufficient range in the types of placements to be able to match to skills and experience of every individual.

All described the three-way meetings between the CM, participant, and line manager as helpful to negotiating work plans. Placement managers rarely contacted the CMs, although CMs thought this may have been because they were contacting the placement managers on a regular basis.

A number of workplace adjustments were negotiated for participants during the initial meetings, and these are outlined in Table 7. No referrals were made out to existing healthcare.

**Table 7:** Commonly negotiated initial workplace adjustments

Making sure people were working at their optimum time of day e.g. when most alert Flexible working (both start and finish times) Providing chairs to sit on when needed Giving breaks Changing work tasks Opportunities to change posture at regular intervals Having designated workstation rather than hot desking Avoiding heavy tasks and lifting Having a named work buddy	

CMs reported that some individuals, especially those with low confidence or high anxiety, required increased time. CMs were employed part-time, and providing flexible management alongside other jobs was reported as difficult at times. CMs used phone, email, and SMS messaging to maintain contact. Overall, time spent on case management was reported as 161.5 h, i.e*.* 125.35 h spent on the 15 placed participants (median = 7.5 (IQR 5.7 to 10) per person) and 45.2 h for the unplaced participants (median = 3.0 (IQR 1.5 to 4.0) per person). In total, our occupational health nurse CM managed five participants (averaging 4.0 h per participant), our employment specialist CM managed 15 participants (averaging 6.7 h per participant), and our stand-in occupational therapist CM managed 10 participants (averaging 4.6 h per participant).

### Participants’ perspectives

We held focus groups with five participants who were matched to placements, three who were not matched to placements, and we held one interview with one person who had been placed but who did not complete the placement. Participants generally reported finding the process of joining and completing paperwork straightforward. Participants were positive about the work preparation session and its facilitator. Feelings of being less isolated, less guilty, and more understood were reported following being in the company of other people with chronic pain and the content (e.g. quotes 11, 12). One noted finding the session more health-focused and tailored to needs than other courses but would have liked more focus on disability.

Participants described regular and supportive contact with CMs; one said they would have liked more contact. One commented specifically on how much they valued the understanding the CM (occupational health nurse) had of their medical condition, in that they had researched it and brought along an NHS leaflet about the condition on the first visit (quote 13).

There were positive views of the placements with participants noting that it allowed them to use their skills and past experience. One commented on being given considerable responsibility because of his previous work experience (quote 14). Others felt they were not entirely suited to the placement they were given and had less opportunity to use their skills. Another commented on how a colleague had made her feel welcome and supported, noting that the placement helped her get back into an active routine, gave her invaluable IT skills, and was a distraction from her pain.

Some of those not placed indicated that they came into the study with high expectations and felt let down. However, some noted still being pleased at having joined the study as they had found the work preparation session helpful and valued the opportunity to meet people in a similar situation (quotes 15, 16).

The participant who left a placement to move into full-time paid employment described an increase in confidence (quote 17), and participants on other placements at other sites were hopeful the placement would lead to opportunities for work. Some felt it would make a difference in terms of getting a job.

Table 8 shows a summary of the key findings relating to feasibility, challenges faced, and lessons learned from across the results sections presented.

**Table 8:** Summary of key findings relating to feasibility, challenges faced, and lessons learned

• It is necessary to allow for an increase in contracting time between the research institution multiple partners. • Matching participants’ skills and aspirations to placement types is difficult in some cases. • It is necessary to ensure a good range of placements up-front, ideally with redundant capacities that match recruitment site areas under the assumption of good recruitment. • It is necessary to ensure early involvement of those who will provide placements and not only high-level management. • Multiple placement providers and recruitment sites are needed. • Employment services sites recruit more participants than NHS pain clinics. • Forming a work plan as a collaborative exercise between participant, case manager, and placement manager works well. • Additional issues surrounding confidence, anxiety, learning difficulties, or comorbidities to chronic pain complicates cases and requires increased case manager time. • Participants value the work preparation session, which facilitates fellowship with those in similar circumstances and removes feelings of isolation. • Some participants find it dispiriting if they are unable to be placed, but these participants still value the work preparation session. • Participants value the support and understanding of the case manager. • Follow-up response rate is relatively poor, and additional effort and consideration may be needed to improve this in a full trial.	

## Discussion

### Main findings and implications

We developed an intervention that was accepted and valued by those to whom it was delivered. We experienced difficulties with recruitment in one location and challenges to providing sufficient placements in another. Either of these factors and particularly the interaction of them both would hinder the implementation of the intervention in a full-scale trial. Substantial improvements to both placement and recruitment rates would be needed to ensure that sufficient participants and placements could be recruited and operated in a full-scale trial.

Risks to poor recruitment could be minimised in a full trial through increasing the number of placement providers and recruitment sites. Our results suggest that employment services yield better recruitment rates than NHS pain clinics; thus, adding additional employment services sites in a full-scale trial may be the most effective remedy. We focused on examining the feasibility of recruiting from pain clinics, as based on our experience from recruiting to studies of low back pain in primary care; we estimated recruitment to be no more than 0.6/1,000 patients in those settings, which would make recruitment from general practice to any subsequent main trial challenging [28].

Risks to maintaining an adequate placement rate could be minimised within a full trial both by securing placements in advance and ensuring a good geographic spread of placements that provides good matched coverage with respect to each recruitment site. Ensuring that during initial engagements with placement providers’ discussions are inclusive of local and placement management, in addition to higher/more central management, may improve the workability and availability of placements to match demand as it is created in the trial.

In our study, our three placement providers had agreed to provide 30 placements, i.e. one for each of our planned participants. While there was a good degree of flexibility within many of our placements, and several alternative placement options were provided, aiming to secure a higher number of placements than planned participants in a full-scale trial would improve the range in the portfolio of placements and facilitate a better matching of placements with participants’ skills and aspirations, which had been a source of dissatisfaction for some in this feasibility study.

Excluding dropouts, response rates for returning outcome measures ranged from 42% at 6 weeks to 53% at 14 weeks, dropping to 47% at 6 months. This could potentially impact on the usefulness of a full trial. The rate is lower than would be expected in trials of interventions to reduce chronic pain, or in work absence trials of interventions for chronic pain, where response rates tend to exceed 70 %[29–32]. At the time of writing, we can find no population like-for-like comparisons. Response rates may be improved in a future trial through reducing the number of outcome measures, and/or using electronic patient reported outcomes (ePROs) with notifications being sent to participants’ devices, possibly through provision of devices to complete these ePROs, and through aiming to collect a very parsimonious minimum set of data in phone calls to non-responders made by well-trained trial managers.

That no referrals to existing health care were needed supports the premise of offering a largely demedicalised intervention. It has been claimed that RTW in those who have been off work with low back pain for more than 2 years is near zero [33–35]. If the developed intervention were to lead to 19% gaining/RTW (the figure observed here) and the proportion gaining/RTW without the intervention is as high as 10%, the resulting number needed to treat of 11 may be attractive for an intervention that requires between four and seven case manager hours per person [36]. Allowing (at the time of writing in the UK) £30 per hour (inclusive of on-costs) for case management that the aspect of the intervention would cost up to £210 per person.

### Comparisons to existing and ongoing research

There is some suggestion that recruitment may be challenging. A trial (NCT02697656) of an IPS work intervention for people in Oslo was designed to recruit 80 participants [37]. However, 65 were reported recruited prior to cessation of recruitment, which unless the trial was stopped in the case of extreme effectiveness, may suggest some difficulties in recruitment [38]. We are also aware of a UK-led pilot study (ISRCTN30094062), in which one of the aims is to explore the feasibility of recruitment of 80 participants from primary care settings, with half being randomised to the intervention. This trial, which is due to end in December 31, 2019, will provide useful comparison to the Oslo-IPS trial and our own work. In addition, the UK government’s joint Work and Health Unit is conducting randomised controlled trials (RCTs) of IPS for people with physical health conditions in the Sheffield City and the West Midlands regions of the UK, across primary, secondary, and community care, and that the results of this trial are due by 2021 [39].

Compared to the IPS approach, a highly specified intervention that provides competitive paid employment and time-unlimited support, our intervention is less intensive and would likely be less expensive. Relative cost-effectiveness would need to be explored in full-scale trials.

The issue of participants’ concerns about losing benefits and the intervention being seen as potentially providing evidence of being fit to work, and therefore potential disqualification from benefits, has been looked at elsewhere. An international evidence synthesis explored how workers’ benefits can act as obstacles to work participation for those with low back pain [40]. They reported finding evidence to suggest that specific unhelpful characteristics of compensatory systems, along with lack of communication/cooperation, are obstacles to work participation, and emphasise a necessity for a ‘whole systems’ approach. This chimes with our experience of trying to deliver our intervention, in which existing infrastructures and communication pathways do not support integrated approaches. Until there is a change, case managers may need to additionally be trained to better understand compensatory benefits systems and how to work practically within local infrastructures.

### Strengths and limitations

Our work provides useful information about the nature of obstacles to RTW in this population and the design of an intervention to help people to overcome these in a practical way. Our work also highlights hazards of recruiting and operating an intervention for this population and aspects that would need addressing prior to doing an RCT. We were able to offer a good range of roles in the Birmingham area, where we had relatively poor recruitment; whereas in Coventry, we experienced relatively good recruitment but had a smaller and less diverse placement range. This meant that we were unable to place as many participants as we had aimed.

Had we been able to execute research contracts with partners earlier, we would have been able to recruit and place more people. Delays may have been in part due to multiple contracts being needed, and there appeared to be limited capacity within the university’s contracting department.

One limitation of our design may have been the use of self-report of attendance at placements to indicate dose received. In a full-scale trial comparison of self-reports and manager reports may aid interpretation. Data on attendance could be requested by the case manager from the placement manager during their regular contacts. Any divergence between the reports could then be reported as results and modelled as appropriate.

We required participants to attend a work preparation session. This effectively adds an inclusion criterion that participants must need to be able and willing to attend that session. It may be argued that this excludes a group of people who with more help might have also participated. We were targeting a population who wanted to work but who perceived that they had obstacles to maintaining sustained employment. We consider that the requirement of attendance at the two-day session approximates the minimum level of ability at which people are likely to benefit from our intervention in practice. While our intervention was open to people who had never worked, we note that all of our participants had previously held employment. The intervention may appeal more to people from this population than one in which employment had not ever been held, although we see no reason to exclude those who had not ever held employment from receiving the intervention in a future trial.

### Recommendations for the future

Further work to explore how to improve recruitment and tackle specific issues such as fear of losing benefits may be beneficial. A well-designed discrete choice experiment may help in achieving this through exploring what intervention attributes potential participants value the most so that interventions can be made more attractive.

Better validation of work-related outcome measures, and subsequent agreement on standardisation of outcomes for trials of interventions to help unemployed people with chronic pain to return to work, would likely facilitate cross-trial comparisons. Our review of IPS-based interventions identified that authors have often defined RTW differently, in some cases with RTW being considered as achieved if a person had done as little as 1one day of work [14]. Sustained work/RTW may be more appropriate as a health outcome in a future trial of this intervention [2, 41]. We agree with Jensen et al., who pragmatically defined the period of RTW as the first consecutive four-week period without sick leave [42].

To allow for contracting time and to help ensure value for money, increased time allocation for contracting should not involve a proportional increase in full-time equivalent allocation to the project, since researcher activities may be dependent on contractual milestones and are limited until contractual execution. Careful design of trial activities and funder-led mechanisms for clawback from universities in the case of long delays may incentivise improvements in performance.

The qualitative feedback in our study suggests that participants found contact with each other valuable. It may be worth exploring cost-effectiveness of a more parsimonious intervention featuring, for example, only a work-preparation course (i.e. a mini-intervention, excluding placements, or ongoing support) and creating more society for people with chronic pain who want to work.

## Conclusions

An intervention that is based on providing six-week individual supported work placements for people who have chronic pain and who are unemployed was acceptable to participants and partners. Trialling the intervention at full-scale could be feasible with careful attention to three main processes. To ensure advanced availability of a sufficiently wide range of work placements in each area, multiple partners from both private and public sectors would be needed to ensure provision of a sufficiently wide range of placements in each area of recruitment. Multiple recruitment sites would be required to gain sufficient recruitment and focusing on employment services will yield better recruitments rates than pain clinics for recruiting participants. Follow-up response rates may be relatively poor and require more effort than usual and/or additional approaches or incentives.

## Supplementary informations

**Additional file 1.** Available and matched placement details. Description: Table detailing placements that were available, including title, type of provider, details of the role, and which placements were filled.

## Data Availability

The datasets used and/or analysed during the current study are available from the corresponding author on reasonable request.

## Abbreviations

CM: Case manager
CMP: Chronic musculoskeletal pain
ePRO: Electronic patient reported outcomes
EQ-5D-5L/3L: EuroQol-5D-5L/3L
HADS: Hospital Anxiety and Depression Scale
IPS: Individual Placement and Support
*n*: Number sampled
NHS: National Health Service
PAW: Psychosocial aspects of work
PPI: Public patient involvement
PROMIS: Patient-Reported Outcomes Measurement Information System
RCT: Randomised controlled trial
RTW: Return to work
SD: Standard deviation
UK: Unitied Kingdom
UNTRAP: University/User Teaching and Research Action Partnership
WRFQ: Work Role Functioning Questionnaire
