# Supplementary material for: Development and feasibility of an intervention featuring individual supported work placements to aid return to work for unemployed people living with chronic pain
Source: Pilot Feasibility Stud. 2020 Apr 22;6:49. doi: 10.1186/s40814-020-00581-6 (PMC7175501; doi:10.1186/s40814-020-00581-6)
Supplement: Supplementary file 1 — Additional file 1. Available and matched placement details. Description: Table detailing placements that were available, including title, type of provider, details of the role, and which placements were filled. [file 40814_2020_581_MOESM1_ESM.pdf]

Additional File 1 – Available and matched placement details

No.	Placement Title	Provider	Details/duties/notes	Location	Filled by
1.	Tender assistant	University of Warwick	Assist procurement team with administrative tasks	Coventry	PP02
2.	Retail and Information Assistant	Council	Working in retail and information centre in a country park	Coventry (outskirts)	PP08
3.	Events and Activities Placement	Council	Part of an events and activities team in a country park	Coventry (outskirts)	PP14
4.	Events and Activities Placement	Council	Part of an events and activities team in a country park	Coventry (outskirts)	PP21
5.	Administrative Assistant	Council	Council logistics and data entry	Coventry	PP11
6.	Assistant Trainer	Council	IT support	Coventry	PP18
7.	Administrative Assistant	Council	Meet and greet, data entry, record keeping, and phones	Coventry	PP09
8	Administrative Assistant	Council	Meet and greet, data entry, record keeping, and phones	Coventry	PP26
9	Patient Advice Liaison Service	NHS Trust	Administrative assistance	Birmingham	PP04
10	Estate maintenance operative	Council	Maintenance within a cemetery and garden of remembrance	Coventry	PP19
11	Estate maintenance operative	Council	Maintenance within a cemetery and garden of remembrance	Coventry	PP24
12	Cleaner	Public Services Provider	Cleaner within a leisure centre	Birmingham	PP22
13	Radiography helper	NHS Trust	Meeting patients and assisting in X-ray departments	Birmingham	PP23

14	Radiography helper	NHS Trust	Meeting patients and assisting in X-ray departments	Birmingham	PP30
15	IT Assistant	NHS Trust	IT/ Audit assistant within IT department	Birmingham	PP20
16	Estate Assistant (withdrawn)	Council	Estate assistant within a country park. Eventually withdrawn due to staffing issues	Coventry	Unused
17	Administrative Assistant	NHS Trust	Administrative assistant within a breast screening unit	Birmingham	Unused
18	Administrative Assistant	NHS Trust	Administrative assistant within a booking unit	Birmingham	Unused
19	Front of House Receptionist	NHS Trust	Administrative assistance at Front of House reception	Birmingham	Unused
20	Nursing Assistant	NHS Trust	Nursing Assistant in neurology	Birmingham	Unused
21	Nursing Assistant	NHS Trust	Nursing Assistant in geriatric medicine	Birmingham	Unused
22	Nursing Assistant	NHS Trust	Nursing Assistant in oncology	Birmingham	Unused
23	Nursing Assistant	NHS Trust	Nursing Assistant in burns	Birmingham	Unused
24	Nursing Assistant	NHS Trust	Nursing Assistant in Accident and Emergency	Birmingham	Unused
25	Theatre Support Worker	NHS Trust	Porting services	Birmingham	Unused
26	Transport Assistant	NHS Trust	Delivering goods and specimens	Birmingham	Unused
27	Physiotherapy Assistant	NHS Trust	A supportive role unless participant had relevant previous experience and qualifications	Birmingham	Unused
28	Housekeeper	NHS Trust	Assisting with cleaning wards and hospital corridors	Birmingham	Unused

29	Porter	NHS Trust	Porting services	Birmingham	Unused
30	Logistics	NHS Trust	Assisting (physically) with receipt and movement of goods	Birmingham	Unused
31	Stores operative	NHS Trust	Storekeeping and movement of goods	Birmingham	Unused
32	Catering Assistant	NHS Trust	Assisting with preparation of food	Birmingham	Unused
33	Medical engineering	NHS Trust	A supportive role unless participant had relevant previous experience and qualifications	Birmingham	Unused
34	Phlebotomy assistant	NHS Trust	A supportive role unless participant had relevant previous experience and qualifications	Birmingham	Unused
35	Administrator	Public Services Provider	Administrative tasks within environmental services	Oldbury	Unused
36	HR Support Co-ordinator	Public Services Provider	Administrative tasks	Bournville	Unused
37	Service Desk Analyst	Public Services Provider	IT support and incident management	Bournville	Unused
38	Catering Assistant	Public Services Provider	Food preparation and serving assistant	King’s Heath	Unused
39	Receptionist	Public Services Provider	Administrative tasks	Perry Barr	Unused
40	Recreation Assistant	Public Services Provider	Setting up equipment, cleaning, and vending machine filling	Acocks Green	Unused
41	Catering Assistant	Public Services Provider	Food preparation and serving assistant	Stechford	Unused
42	Recreation Assistant	Public Services Provider	Supporting the operation of the site, includes setting up sports equipment, basic cleaning tasks, and filling vending machines	Stechford	Unused

43	Canteen Assistant (withdrawn but offer to replace with additional HR/IT roles as per 36/37)	Public Services Provider	Preparing sandwiches, serving customers, writing sandwich labels, re-stocking fridge.	Bournville	Unused
